# Structural Factors Inducing Cracking of Brass Fittings

**DOI:** 10.3390/ma14123255

**Published:** 2021-06-12

**Authors:** Lenka Kunčická, Michal Jambor, Adam Weiser, Jiří Dvořák

**Affiliations:** Institute of Physics of Materials, Czech Academy of Sciences, 61662 Brno, Czech Republic; jambor@ipm.cz (M.J.); aweiser@ipm.cz (A.W.); dvorak@ipm.cz (J.D.)

**Keywords:** brass, cracking, FEM, scanning electron microscopy, transmission electron microscopy, microhardness

## Abstract

Cu–Zn–Pb brasses are popular materials, from which numerous industrially and commercially used components are fabricated. These alloys are typically subjected to multiple-step processing—involving casting, extrusion, hot forming, and machining—which can introduce various defects to the final product. The present study focuses on the detailed characterization of the structure of a brass fitting—i.e., a pre-shaped medical gas valve, produced by hot die forging—and attempts to assess the factors beyond local cracking occurring during processing. The analyses involved characterization of plastic flow via optical microscopy, and investigations of the phenomena in the vicinity of the crack, for which we used scanning and transmission electron microscopy. Numerical simulation was implemented not only to characterize the plastic flow more in detail, but primarily to investigate the probability of the occurrence of cracking based on the presence of stress. Last, but not least, microhardness in specific locations of the fitting were examined. The results reveal that the cracking occurring in the location with the highest probability of the occurrence of defects was most likely induced by differences in the chemical composition; the location the crack in which developed exhibited local changes not only in chemical composition—which manifested as the presence of brittle precipitates—but also in beta phase depletion. Moreover, as a result of the presence of oxidic precipitates and the hard and brittle alpha phase, the vicinity of the crack exhibited an increase in microhardness, which contributed to local brittleness.

## 1. Introduction

Given their superior characteristics—such as excellent corrosion resistance, exceptional electric and thermal conductivity, non-magnetism, and favourable machinability—Cu-based materials are popular in industry and commerce [1]. Cu is used in numerous forms. For example, commercially pure Cu featuring exceptional electric conductivity is advantageously applied in conductors [2,3]; Cu-based alloys and compounds are primarily used for their high resistance to corrosion in multiple solutions [4]; and modern Cu composites, which can be advantageously combined with polymers or other elements and alloys, provide a combination of light weight and reasonable strength and conductivity (e.g., styrene–ethylene/butylene–styrene, aluminium, iron, etc. [5,6,7,8,9,10,11,12]).

Brasses (Cu–Zn–X), which are produced by adding Pb to the basic elements (Cu and Zn), are probably the most popular Cu alloys. The overall Zn content influences not only the structure and present phases—brasses with very high and very low Zn content feature the beta or alpha phase, respectively [13], while brasses featuring Zn content between 40 and 60 wt.% are biphasic, i.e., they consist of combinations of both the alpha and beta phases [14]—but also the properties of the final brass, such as formability, mechanical properties, corrosion resistance, etc. Brass dezincification, influencing the corrosion resistance, is closely related to its structure, which is primarily determined by its chemical composition. The most economical way to minimize this phenomenon is to add low levels of specific alloying elements, e.g., Al, As, Sn, Sb, B, or P [15,16]. One of the main research tasks for brasses is to improve their machinability; the most favourable machining behaviour was reported for brasses featuring Pb contents lower than 3 wt.%, since Pb has very little solubility in Cu and forms fine precipitates in the final biphasic structures [17]. Nevertheless, heat treatment at temperatures between 400 °C and 600 °C, with subsequent quenching, can favourably modify the structures to be monophasic (alpha phase) [18].

Leaded brass is the material that is primarily used to manufacture gas valves, the production process of which is rather complicated (consisting of casting, followed by extrusion, hot forging, cold drawing, and finally, machining). The deformation behaviour of leaded brasses has been investigated by several research teams, e.g., Suárez et al. [19] analysed the deformation behaviour of a Cu40Zn2Pb brass at high temperatures; Zhu et al. [20] optimized the processing conditions via analysis of processing maps for a Cu25Zn brass; and Mapelli et al. [21] documented the relationships between the textures and morphologies of the present phases and the mechanical properties within a hot-extruded Cu39Zn2.6Pb biphasic brass. Nevertheless, the complicated production process, consisting of multiple steps, can lead to the introduction of undesirable structural defects, which can eventually result in failure (i.e., occurrence of cracking) and decreased longevity of the final product. For example, casting can impart pores, cavities, surface defects, and segregations; extrusion and cold drawing can induce tearing or chevron cracking; hot forging can lead to hot forging laps or flash cracking; and last, but not least, machining can result in lathe jaw marks or thin surface deflection steps [22]. Studies focusing on the elimination of the risk of crack formation during processing of brasses have been published. The Pb content has been found to affect chip formation during machining, as lead forms islands of precipitates at the interfaces of both the alpha and beta phases [23]. Heat treatment at 775 °C for 60 min was shown to improve the fracture toughness of lead-free brasses [24]. Other studies used heat treatment to optimize the structure parameters, in order to favourably affect the morphology of the grains and the final properties of a 60/40 brass [25], and investigated the relationship between the processing technology of a leaded brass and its final mechanical properties and structures [26].

The research performed so far has documented that structure morphology—and grain size in particular—is the primary factor influencing the mechanical properties and final performance of brass products. The lower the grain size, the more positive the effect on the final properties, as also proven by a recent study documenting the effects of the equal channel angular pressing (ECAP) method on the mechanical properties of a brass [27]. ECAP is one of the severe plastic deformation (SPD) methods, which are based on imparting severe shear strain onto materials, with the aim of introducing significant grain refinement—to the ultra-fine (UF) or nano scale—to enhance the final properties [28,29,30,31,32,33,34,35,36]. Nevertheless, as far as the authors’ knowledge reaches, no study reporting the correlation of structure with defects occurring/developing within a real brass gas valve component produced under industrial processing conditions has been published thus far. Therefore, the focus of the present paper is to find any correlation between the real two-stage production process (i.e., hot plastic deformation process) of a fitting—i.e., a pre-shaped medical gas valve made of Cu40Zn2Pb brass—and the occurrence of failure in a specific location of the component. Numerical simulation was implemented primarily to analyse the plastic flow during hot forging, and to correlate this factor with the results of predicted stress distribution during forging, since locations with increased stress values or inhomogeneous stress distribution are the locations with increased probabilities of the development of defects.

## 2. Materials and Methods

**Figure 1:**
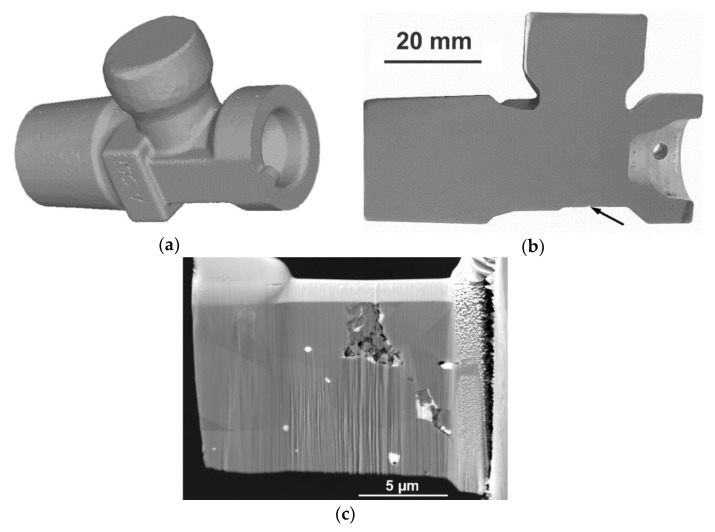
Numerical model of the investigated brass fitting (**a**); real cut through the brass fitting, with the location of cracking marked (**b**); lamella of the cracked region (before final milling), acquired using FIB (**c**).

The aim of the work was to carry out detailed characterization of the structure of a brass fitting, with the focus on a specific location that exhibited cracking after hot deformation processing. The fitting was produced by Gas Control Equipment (GCE) (Chotěboř, Czech Republic) from the CuZn40Pb2 alloy. Figure 1a shows a model of the entire fitting created by the finite element method (FEM), using Forge NxT^®^ software, whereas Figure 1b depicts the longitudinal axial cut through the real fitting, with the location of cracking marked. The original brass rod—i.e., the semi-product for the fitting—was fabricated via casting, followed by direct extrusion. The rod was subsequently induction heated, and eventually hot die-forged to produce the final fitting. The first analysis performed after processing was verification of the chemical composition using an inductively coupled plasma atomic emission spectroscope (ICP-AES, SPECTRO ARCOS II device by SPECTRO CS, spol. s r.o., Ostrava, Czech Republic). The results of this analysis are summarized in Table 1; the measured overall contents of the individual elements were in accordance with the ČSN 42 1300 standard, as required by the producer.

The following analyses, for which a digital optical microscope (Olympus DSX1000, Tokio, Japan) was used, focused on the characterization of plastic flow during die forging. For detailed structure observations, we used a Lyra 3 XMU scanning electron microscope (SEM, Tescan, Brno, Czech Republic) and a JEM-2100 transmission electron microscope (TEM, JEOL, Tokio, Japan) operating at 200 kV. A symmetry electron backscatter diffraction (EBSD, Oxford Instruments, Abingdon, UK) detector attached to the SEM was applied in order to examine the present structure’s phases, whereas an Ultim Max energy-dispersive spectroscope (EDS, Oxford Instruments, Abingdon, UK) equipped on the TEM was used for detailed characterization of the local chemical compositions. The analysed samples involved a cracked fitting cut longitudinally along its axis (see Figure 1b), as well as individual SEM and TEM samples of the cracked fitting. Preparation of the samples was performed by the combination of manual grinding and polishing on SiC papers and colloidal silica. The sample prepared for SEM-EBSD analysis was subjected to scanning with a 1 µm scan step under a tilt of 70°; the scans were evaluated using the AZtec Crystal software (Oxford Instruments, Abingdon, UK). The focused ion beam (FIB) technique of the Tescan Lyra 3 XMU device was further used to extract a lamella for the TEM observations. Using FIB, a thin protective Pt layer was first deposited on the original surface of the examined sample. Subsequently, the lamella was milled with 30 keV Ga ions to a thickness of ~150 nm (see Figure 1c for the lamella before the final milling). Finally, fine milling with 5 keV Ga ions was used to achieve the final thickness of the lamella of ~100–120 nm.

The structure study was supplemented with measurements of Vickers microhardness (in HV, Zwick/Roell testing machine, Zwick Roell CZ s.r.o., Brno, Czech Republic) along the cracked surface and in the vicinity of the cracked area, as well as in the die-forged material exhibiting no failure (the internal area and surface area of the fitting exhibiting no defects). The loading time for the measurements was 10 s, and the load for each indent was 200 gf. To analyse the microhardness in the internal and surface areas with no failures, 10 indents were randomly executed in particular locations of the brass component, the average values of which were subsequently calculated.

Last, but not least, numerical simulation of hot die forging using FEM was performed with the help of Forge NxT^®^ software (Transvalor s.a., Biot, France). The simulation was carried out with an assembly with geometrical dimensions and mechanical properties identical to those of the real components. The dies were considered to be rigid, whereas the brass billet was meshed with tetrahedral elements. Based on the real industrial conditions, the defined dies’ speed was *v* = 3 mm/s, and graphite-based lubricant was applied. The defined boundary conditions, applicable at the elevated forging temperature—i.e., 680 °C—were the Young’s modulus (30 GPa), Poisson’s ratio (0.35), thermal expansion coefficient 2.2 × 10^−5^ (K^−1^), thermal conductivity (120 (W/(mK)), specific heat (380 J/kgK), emissivity (0.7), and density (8440 kg/m^3^). The parameters of the simulations were defined via the elastic–plastic model, with the Newton–Raphson convergent algorithm, while the deformation behaviour of the brass billet was characterized via the Hansel–Spittel equation (Equation (1)):

(1) $$\sigma_{f}=Ae^{m_{1}T}T^{m_{8}}\varepsilon^{m_{2}}e^{m_{4}/\varepsilon }{\left(1+\varepsilon \right)}^{m_{5}T}e^{m_{6}\varepsilon }{\dot{\varepsilon }}^{m_{3}}{\dot{\varepsilon }}^{m_{7}T}$$

where ε is equivalent strain, *T* is temperature, ε is equivalent strain rate, and *A* and *m*_1_ to *m*_8_ are regression coefficients, the values of which are: *A* = 8039.59 MPa, *m*_1_ to *m*_4_ = −0.00835, −0.00099, 0.1693, and −0.00924, respectively, and *m*_5_–*m*_8_ = 0. 

## 3. Results

### 3.1. Finite Element Analyses

**Figure 2:**
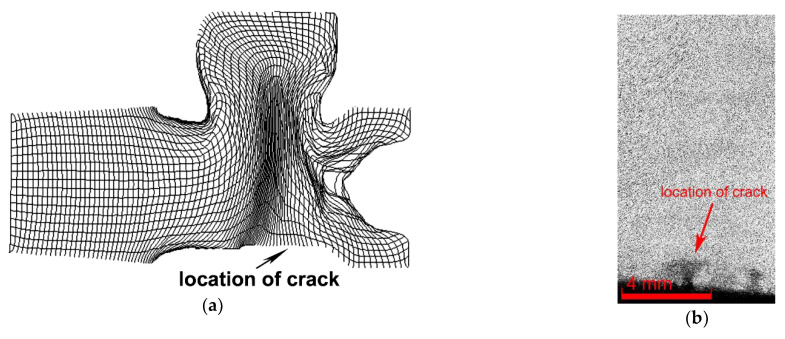
Plastic flow along the axial longitudinal cut through the brass fitting acquired via numerical simulation (**a**); detailed OM view of plastic flow in the vicinity of the crack within the real component (**b**).

Numerical predictions and optical microscopy (OM) were the primary instruments used to analyse the plastic flow of the brass rod during the die forging of the fitting. Figure 2a shows a mesh superimposed through the axial longitudinal cut through the brass fitting, depicting the plastic flow of the material during the hot die-forging process. The supplementary detailed OM view of the flow of the grains along the axial longitudinal cut through the component (the crack can be seen in the bottom right corner) is depicted in Figure 2b. Both the figure from the simulation and the one from real forged part show that the plastic flow was aggravated in the area in which the crack occurred and in its vicinity, as the grains of the forged brass rod—the axis of which was parallel to the longitudinal axis of the fitting—primarily flew into the upper socket of the fitting, i.e., in the very opposite direction to the bottom horizontal area of the fitting in which the cracking finally occurred. The plastic flow also exhibited local instabilities, which manifested as a severely distorted grid in those particular locations (see Figure 2a).

**Figure 3:**
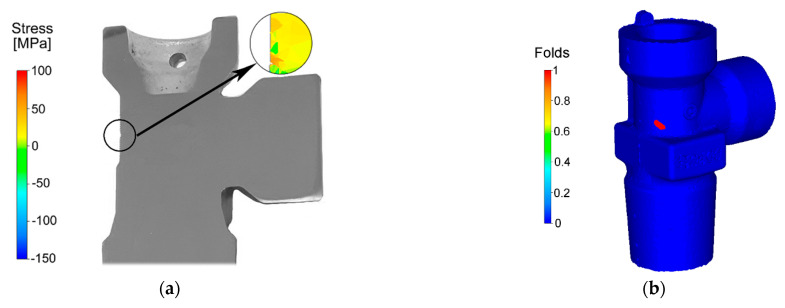

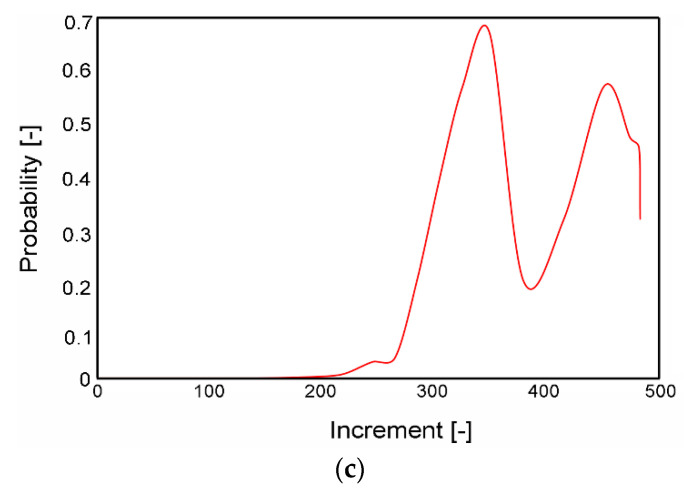
Stress distribution during hot forging in the location of the crack (**a**); predicted probability of the occurrence of folds in scale from 0 (0%) to 1 (100%) (**b**); Cockroft–Latham criterion during the die-forging processing step (**c**).

Figure 3a depicts the numerically predicted stress distribution in the location in which the crack occurred on the axial longitudinal cut through the fitting during hot forging. The prevailing stress in the cylindrical part of the fitting was of a compressive character. However, the part of the fitting featuring complex geometry exhibited the majority of the tensile stress, the value of which generally increased towards the shaped areas of the brass component. Nevertheless, certain locations on the fitting featured local stress inhomogeneities and combinations of compressive and tensile stresses, as can be seen in the circle in Figure 3a.

As regards the energy imposed on the brass semi-product via the plastic deformation, its distribution was more or less inhomogeneous, as documented by the parameter of the probability of the nucleation of forging defects, the distribution of which on the surface of the forged fitting is depicted in Figure 3b. As can be seen, the location in which this parameter was the highest corresponded to the location on the real fitting in which the defect occurred (see Figure 1b and Figure 3a). Subsequently, the probability of the occurrence of failure in this particular location was evaluated via the Cockroft–Latham criterion, since evaluation of this criterion is especially beneficial for the presence of tensile stress in a particular location (as documented in Figure 3a). As evident from Figure 3c, the probability of the nucleation of defects increased rapidly approximately halfway through the processing step, and the criterion reached its maximum value of approximately 70% in the moment at which the upper socket of the fitting started to form (i.e., increment 350).

### 3.2. Structure Analyses

**Figure 4:**
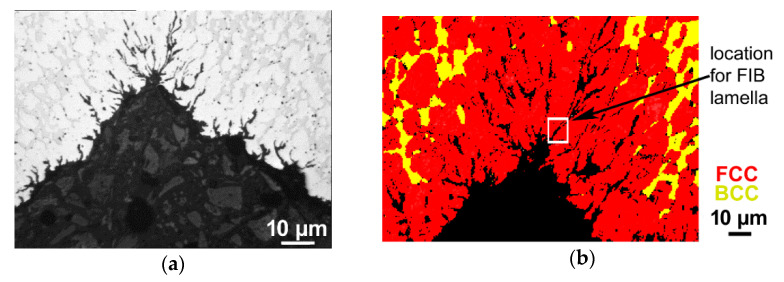
OM image of the cracked area (**a**); SEM-EBSD scan of the cracked area, with a map of phase composition (**b**).

Subsequent analyses primarily focused on the structural factors in the vicinity of the crack, which could have contributed to the observed failure of the brass fitting. Figure 4a depicts a detailed optical microscopy image of the cracked area. As can be seen, the defect consisted of a stem-like branched crack, which developed at the surface of the brass component and proceeded towards its internal region in a direction more or less perpendicular to the longitudinal axis of the fitting. 

OM did not reveal any essential information about structural anomalies; in other words, no evident differences in the grain sizes were detected. The primary hypothesis was thus that the cracking occurred due to local changes in the phase composition or chemical composition. Therefore, SEM-EBSD was further applied in order to acquire detailed information about the present phases in the cracked location of the fitting.

Figure 4b shows an SEM-EBSD scan depicting the phases present in the cracked area of the fitting; the FCC (face-centred cubic) alpha phase is depicted in red, whereas the BCC (body-centred cubic) beta phase is depicted in yellow. The figure clearly shows that the surface of the fitting, as well as the entire cracked area, were depleted of the beta phase, as only the alpha phase was detected at the surface of the die-forged component.

**Figure 5:**
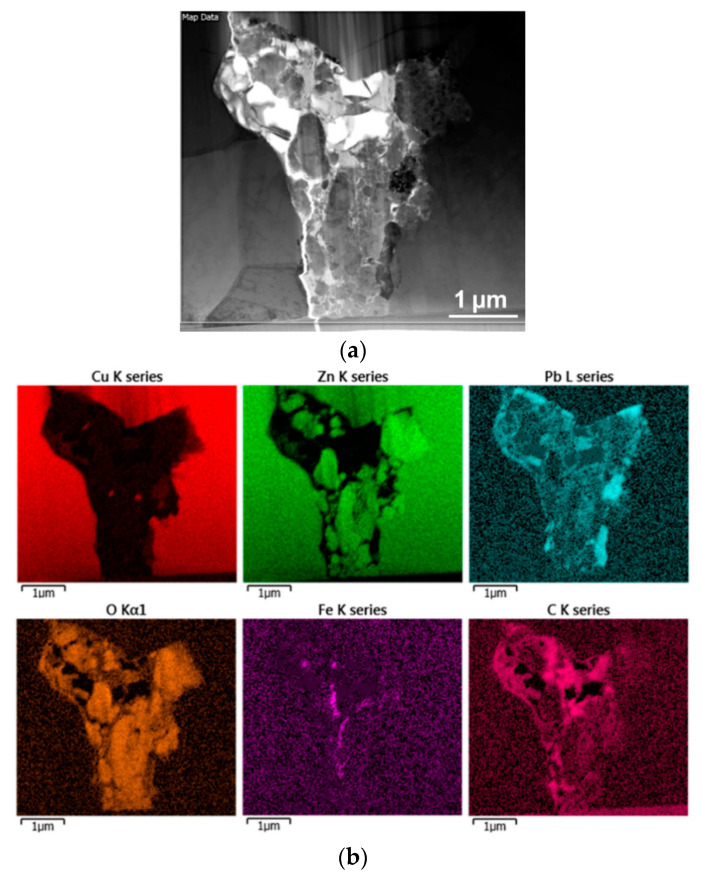
TEM scan (bright field) of the examined location with depicted area of EDS mapping (**a**); EDS maps for individual elements for the scanned area (**b**).

The structure analyses performed via SEM showed differences in the phase composition (see Figure 4b). Nevertheless, they did not reveal detailed information about the presence of possible precipitates or intermetallic phases in the vicinity of the crack. For this purpose, we prepared a lamella via FIB (see Figure 1c for the lamella and Figure 4b for the location from which the lamella was collected) and applied TEM. Firstly, we used TEM-EDS mapping to characterize the overall presence and distribution of the individual elements in the vicinity of the crack. The scanned area is depicted in Figure 5a, and the corresponding EDS maps for the individual elements are depicted in Figure 5b. As can be seen from the maps, the area surrounding the crack consisted mostly of Cu, with additional Zn and Pb, as expected. The area of the crack then exhibited negligible Cu content, but increased concentrations of Zn, Pb, O, C and, locally, Fe. Subsequent investigations were focused on detailed examinations of the locations (particles) of interest.

**Figure 6:**
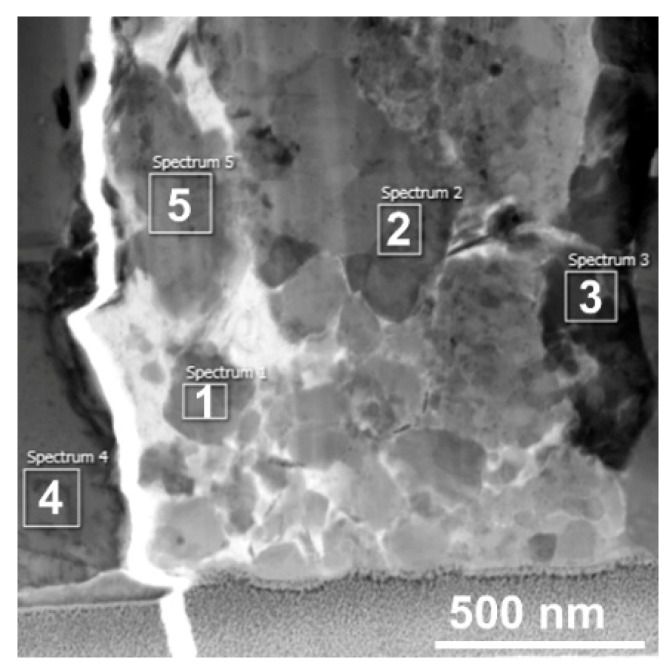
TEM scan (bright field) of the detailed view of the cracked location, with depicted areas of local EDS scanning.

Figure 6 depicts a more detailed view on the area of the crack, in which subsequent analyses of the locations of interest were performed. The figure shows five individual locations (spectra) that were scanned for their detailed chemical compositions. The results of the scanning are summarized in Table 2. As can be seen from the table, the scan acquired from the location next to the crack—i.e., scan 4—exhibited only the presence of the brass elements characterized in Table 1—i.e., Cu, Zn, Pb, and Si. Scan 3—i.e., the location in very close proximity to the crack—also exhibited the presence of the basic brass elements, but together with quite a high amount of oxygen. The scans acquired from the particles present in the crack—i.e., scans 1, 2, and 5—all exhibited very little presence of Cu, but high content of Zn and/or Pb and Fe, together with oxygen (the presence of Na was most likely a remnant from the preparation of the samples).

### 3.3. Microhardness

**Figure 7:**
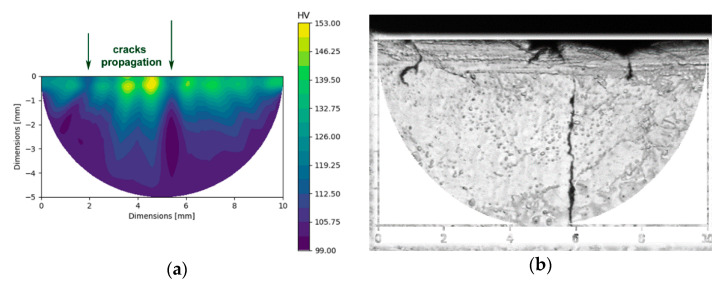
Map of experimentally measured microhardness in the cracked area (**a**); the corresponding cracked location, with depiction of the mapped area (**b**).

The detailed structural analyses were supplemented with analyses of microhardness in the internal and surface areas of the examined fitting, as well as in the vicinity of the cracked location. The investigations showed that the average HV value measured in the internal area of the fitting was 99 HV, whereas the average HV value measured at the surface of the fitting was almost 114 HV. The surface area in the closest proximity to the cracked location exhibited even higher microhardness; its average value in this area was 132.5 HV. Figure 7a depicts the map of microhardness acquired in the area of cracking—i.e., the area corresponding to the OM image depicted in Figure 7b (Figure 7b contains a schematic depiction of the region of the HV map, clearly showing the real area for which the microhardness was measured).

The images demonstrate that the microhardness was increased along the cracked surface, which resulted in decreased ductility, local brittleness, and eventual development of cracks. The map also shows that the occurrence of cracks consequently decreased the microhardness values along the cracks (see the local decreases in the HV values in the directions of the arrows depicted in Figure 7a, corresponding to the actual cracks in Figure 7b).

## 4. Discussion

We primarily performed the herein-presented analyses in order to investigate structural factors and anomalies, which could have caused the occurred cracking of the provided brass fitting—i.e., the pre-shaped medical gas valve. The component was fabricated in a rather complicated multiple-step production process. Nevertheless, the final deformation step of hot forging was crucial for the quality of the brass valve, as the material was subjected to substantial plastic deformation at a relatively high processing temperature (~680 °C). 

Before forging, the brass semi-product was heated via induction. Based on the conclusions of published studies documenting the importance of having optimized the parameters of induction heating before the actual heating preceding the plastic deformation [37], we performed numerical simulations, with the focus on prediction of the selected parameters, in order to evaluate the suitability of the designed production process. The predicted stress distribution revealed that the die-forged brass did not exhibit any significant local peaks of either tensile or compressive stress values. On the other hand, the stress distribution exhibited local inhomogeneities, one of which was evident in the location of the crack. The stress inhomogeneity, as well as the inhomogeneity of the imposed energy, can be primarily attributed to the geometry of the forging die.

The numerical analysis of plastic flow revealed instability of material flow in the area in which the material flew into the upper socket of the fitting (see Figure 1b for the geometry of the fitting and the exact location of the crack, and Figure 2a for the predicted plastic flow), and also showed aggravation of the plastic flow in the location of the crack. This phenomenon was most likely induced by, among other factors, the increased friction of the forged brass with the die in this location [38]. Plastic flow instabilities also increase the risk of the occurrence of folds, as seen in Figure 3b,c. The experimental analysis of plastic flow performed via OM showed differences in the plastic flow in the individual locations of the fitting as well. The real material flow within the original extruded brass rod—the horizontal axis of which was parallel to the longitudinal axis of the brass fitting during die forging—was primarily towards the upper socket of the fitting. The aggravated flow of the material in the location of the crack most likely contributed to the observed defect, especially when considering its mutual effect with other factors—such as friction between the material and the die, or heat transfer through the die—which are important when evaluating the failure of materials, e.g., via computational simulations [38,39]. We also looked for any visible differences in the grain sizes between the internal and surface regions of the fitting. However, OM did not reveal any significant differences in this parameter throughout the structure of the component. The primary hypothesis was thus that the cracking occurred as a result of the mutual detrimental effects of aggravated plastic flow, local stress inhomogeneity, and local changes in the chemical or phase compositions.

As evident from Figure 4b—which depicts the phase composition in the area in the vicinity of the crack, as acquired by the SEM-EBSD method—the surface of the fitting exhibited beta phase depletion. As the beta phase is the high-temperature brass phase [40], this finding is consistent with the previously discussed results. The locally aggravated motion of the material in the die, together with the continuous heat transfer between the die and hot brass during forging, contributed to accelerated cooling of the surface of the forged product, which facilitated the formation of the alpha phase on the surface of the fitting. In biphasic brasses, the beta phase ensures the plasticity and ductility of the material, while the alpha phase provides greater hardness and strength [40]. Therefore, the prevailing presence of the alpha phase decreased the ductility of the material in the location of cracking. This factor consequently also influenced the microhardness, which was highest at the surface where the cracking occurred. On the other hand, crack propagation consequently weakened the material, which resulted in decreased microhardness values in the exact locations of the cracks (see Figure 7a,b). The detailed TEM characterization revealed that the area of the crack contained particles with increased oxygen contents, which points to the presence of oxides. Oxidic particles, especially when they are coarse and behave like interstitials, tend to be brittle and decrease the cohesive toughness of the material [41]. This phenomenon was demonstrated by the microhardness values, which were the highest along the surface on which the crack propagated.

Based on these findings, we can summarize that the mutual interaction of the mentioned factors—i.e., unfavourable stress distribution in the cracked location (most likely developed by the effect of accelerated heat transfer, geometry of the forging die, and corresponding material plastic flow), together with the increased probability of the development of folds and high values of the Cockroft–Latham criterion, in conjunction with the presence of oxides and depletion of the beta phase—was the overall cause for the failure of the brass component. In other words, all of these factors are responsible for the nucleation of cracks and subsequent occurrence of forging defects. Similar conclusions have also been drawn previously for the deformation processing of other metallic materials—such as aluminium [42], composites [43], laminates [44], or tungsten-heavy alloys [45]—for which quite a high level of residual stress was observed due to non-homogenous deformation processing. Although the presence of residual stress within the structure was not determined in the present study, the detrimental effect of this parameter on the occurrence and propagation of the observed crack can be supposed with a high probability, and should be investigated in our next paper.

## 5. Conclusions

The present study primarily focused on the analyses of the structural factors behind cracking in hot die-forged brass fittings—i.e., pre-shaped medical gas valves. The experimental results were supplemented with the results of numerical predictions. Based on the performed analyses, the cracking occurred in a location that exhibited plastic flow instability and inhomogeneity of stress distribution during forging, as well as high values of the Cockroft–Latham criterion, indicating increased probability of the development of defects. Moreover, the surface of the fitting exhibited depletion of the ductile beta phase, which resulted in an increase in microhardness and local brittleness. The location of the crack also exhibited the presence of brittle oxidic precipitates, which also contributed to the failure of the component. Suffice to say, cracking of hot die-forged non-ferrous products can generally be induced by the mutual effects of processing factors—such as the presence of stress and plastic flow inhomogeneities introduced by die geometry—and structural phenomena—such as local changes in chemical/phase composition and the presence of segregated/precipitated particles, which are typically introduced by local inhomogeneities in processing temperature.

## Figures and Tables

**Table 1:** ICP-AES analysis results of the chemical composition of the fitting.

Element	Cu	Zn	Pb	Fe	Sn	Ni	Al	Si	Cd
(wt.%)	58.10	39.60	1.75	0.27	0.20	0.07	0.004	0.003	0.002

**Table 2:** Chemical compositions of the scanned areas depicted in Figure 6.

Element [wt.%] Area Number	Cu	Zn	Pb	Fe	O	Na	Si
1	5.59	43.76	0.22	0.44	40.15	9.46	0.38
2	5.04	46.30	0.25	0.61	39.24	8.23	0.33
3	34.56	1.3	36.97	-	27.18	-	-
4	67.36	31.69	-	0.31	-	-	0.65
5	5.92	44.02	0.36	0.41	41.07	7.72	0.51

## Data Availability

The original data supporting the research is not publicly available but the data that is not confidential is available on request from the corresponding author.
